# 1228. Evaluation of the Practice of Switching from Intravenous to Oral Antimicrobial Therapy at King Abdulaziz University Hospital

**DOI:** 10.1093/ofid/ofad500.1068

**Published:** 2023-11-27

**Authors:** Reem Alharbi, Nour Baghdady

**Affiliations:** King Abdulaziz University Hospital, Jeddah, Makkah, Saudi Arabia; Clinical Pharmacy Department, Faculty of Pharmacy, King Abdulaziz University, Jeddah, Makkah, Saudi Arabia

## Abstract

**Background:**

Conversion from intravenous (IV) antibiotics to an oral (PO) in eligible patients is an important antimicrobial stewardship strategy. Recently, King Abdul-Aziz University Hospital (KAUH) in updated its IV to PO conversion policy. This policy describes eligibility criteria for patients to be switched to oral therapy from IV and it suggests PO alternative to IV medications. The aim of this prospective observational study is to observe IV to PO conversion practices on selected antimicrobials in selected ward after updating and implementing this policy.

**Methods:**

Adult patients admitted to the medical, surgical, and hematology/oncology wards who received either metronidazole, ciprofloxacin or clindamycin IV were evaluated for antibiotic need and for eligibility for IV to PO conversion according to the policy. Evaluation period spanned from November 2021 – December 2022.

**Results:**

194 patients were evaluated. Of them, 154 (80%) had a clear indication for the antibiotic of which 98 (63%) required IV administration for the following reasons: impaired oral route (59%), impaired gastrointestinal absorption (15.3%), acute treatment phase of the infection (15.3%), and other reasons such as surgical prophylaxis (3%) and severe sepsis/septic shock (2%). The remaining 56 (36.7%) met the policy’s eligibility criteria for IV to PO conversion.

**Conclusion:**

We found that most patients received antibiotics appropriately in the appropriate route for an existing indication. However, there are plenty of opportunities for improvement in discontinuing unnecessary antibiotics and de-escalating from IV to PO. Ideally, this discussion should be had while rounding on the patient for collective decision making at the point of care.

**Disclosures:**

**Nour Baghdady, PharmD, BCPS, MPH, BCIDP**, CSL Seqirus: Advisor/Consultant|CSL Seqirus: Stocks/Bonds

